# Co-administration of sodium hydrosulfide and tadalafil modulates hypoxia and oxidative stress on bladder dysfunction in a rat model of bladder outlet obstruction

**DOI:** 10.1590/S1677-5538.IBJU.2022.0207

**Published:** 2022-08-20

**Authors:** Didem Yilmaz-Oral, Ecem Kaya-Sezginer, Heba Asker, Serap Gur

**Affiliations:** 1 Cukurova University Faculty of Pharmacy Department of Pharmacology Adana Turkey Department of Pharmacology, Faculty of Pharmacy, Cukurova University, Adana, Turkey; 2 Ankara University Faculty of Pharmacy Department of Pharmacology Ankara Turkey Department of Pharmacology, Faculty of Pharmacy, Ankara University, Ankara, Turkey; 3 Ankara University Faculty of Pharmacy Department of Biochemistry Ankara Turkey Department of Biochemistry, Faculty of Pharmacy, Ankara University, Ankara, Turkey; 4 Lokman Hekim University Faculty of Medicine Department of Medical Pharmacology Ankara Turkey Department of Medical Pharmacology, Faculty of Medicine, Lokman Hekim University, Ankara, Turkey

**Keywords:** Prostatic Hyperplasia, Urinary Bladder, Overactive, Hydrogen Sulfide, Tadalafil

## Abstract

**Purpose:**

This study aimed to assess the possible healing effect of combination treatment with a hydrogen sulfide (H2S) donor, sodium hydrosulfide (NaHS) plus tadalafil on partial bladder outlet obstruction (PBOO)-induced bladder dysfunction.

**Materials and Methods:**

A total of 75 male Sprague-Dawley rats aged 10-wk and 300-350g were divided into five groups; control; PBOO; PBOO+NaHS (5.6mg/kg/day, i.p., 6-wk); PBOO+tadalafil (2mg/kg/day, oral, 6-wk) and PBOO+NaHS+tadalafil. PBOO was created by partial urethral ligation. 6 weeks after obstruction, the in vitro contractile responses of the detrusor muscle and Western blotting, H2S and malondialdehyde assay were performed in bladder tissues.

**Results:**

There was an increase in bladder weight(p<0.001) and a decrease in contractile responses to KCl (p<0.001), carbachol (p<0.01), electrical field stimulation (p<0.05) and ATP (p<0.001) in the detrusor smooth muscle of obstructed rats which was normalized after the combination treatment. Cystathionine γ-lyase and cystathionine β-synthase, and nuclear factor kappa B protein levels did not significantly differ among groups. The obstruction induced decrement in 3-mercaptopyruvate sulfur transferase protein expression(p<0.001) and H2S levels(p<0.01) as well as increment in protein expressions of neuronal nitric oxide synthase (NO, p<0.001), endothelial NOS (p<0.05), inducible NOS(p<0.001), hypoxia-inducible factor 1-alpha (p<0.01), and malondialdehyde levels (p<0.01), when combined treatment entirely normalized.

**Conclusions:**

Combination therapy has beneficial effects on bladder dysfunction via regulating both H2S and nitric oxide pathways as well as downregulation of oxidative stress and hypoxia. The synergistic effect of H2S and nitric oxide is likely to modulate bladder function, which supports the combined therapy for enhancing clinical outcomes in men with BPH/LUTS.

## INTRODUCTION

Lower urinary tract symptoms (LUTS), including storage, voiding and postmicturition symptoms, are widespread clinical conditions in older men and concurrent with bladder outlet obstruction (BOO) as a consequence of benign prostatic hyperplasia (BPH) (1). Tadalafil is the only phosphodiesterase type-5 inhibitor (PDE5i) approved for treating moderate to severe LUTS/BPH (2). A previous study showed that tadalafil improved blood flow of the bladder as well as functional and histological alterations in the partial BOO (PBOO) rat bladder (3).

A gasotransmitter, hydrogen sulfide (H_2_S) is endogenously synthesized by cystathionine β-synthase(CBS), cystathionine γ-lyase(CSE) and 3-mercaptopyruvate sulfurtransferase(3-MST) (4). H_2_S has various roles, including neuromodulation, smooth muscle cell regulation with its antioxidant activity and anti- or pro-inflammatory effects (5). H_2_S displays a species‐specific difference or diverse site-specific effects on bladder contractility (5). Studies reported that H_2_S and its donors induced relaxation in the human and rat bladder (6, 7).

The precise mechanisms leading to improving pathological components in LUTS remain unclear. Previous data suggest that obstruction leads to structural and functional changes, including urodynamic parameters (8). Furthermore, numerous studies assessed the effects of PBOO on morphologic changes and physiologic mechanisms in the bladder without *in vivo* urodynamic data (9, 10).

Previous data revealed that PDE5i could act through several mechanisms to suppress LUTS. It has also been demonstrated that H_2_S was an endogenous inhibitor of PDE (11). In addition, a PDE5i, sildenafil relaxes human bladder strips and, at similar doses, induces H_2_S production in a concentration- and time-dependent manner (7).

We hypothesized that the improvement in the nitric oxide(NO)/cGMP and H_2_S signaling could ameliorate bladder dysfunction secondary to the obstruction. This study is aimed to elucidate the effects of either a donor of H_2_S, sodium hydrogen sulfide (NaHS) or a PDE5i, tadalafil treatment alone or in combination on bladder dysfunction in rats with obstructed bladder.

## MATERIALS AND METHODS

### Animals

All experimental procedure of the animals was approved by the Ethics Committee of Ankara University(approval no:2015-16-184). Rats were housed in separate cages and provided food and water ad libitum in a temperature-controlled room(22±1°C) artificially lit from 7:00 a.m. to 7:00 p.m. daily.

Male Sprague-Dawley rats(n=75, 10wk, 330.9±3.1g) were obtained from Bilkent University (Ankara, Turkey). Rats were divided into five groups;1:control, 2:PBOO, 3:PBOO+NaHS, 4:PBOO+tadalafil and 5:PBOO+NaHS+tadalafil. After the operation, each rat was treated with NaHS(300μL, 5.6 mg/kg/day) (12) or/and tadalafil(300μL, 2mg/kg/day) (3, 13) once a day for 6wk. Tadalafil tablets(Cialis™ 20mg film-coated tablets, Eli Lilly and Company, Indianapolis, USA) were crushed, freshly suspended in water as a vehicle, and administered orally by gavage feeding after surgery. A total of 5.6mg/kg NAHS(diluted in saline(0.9%); Sigma-Aldrich) was intraperitoneally injected after surgery. The body weights of all rats were calculated via a precision scale before the sacrifice of animals. The bladder and prostate tissues were excised and weighted by an electronic scale after sacrifice.

### Surgical induction of PBOO

Obstruction was induced based on a previous study (14). After a longitudinal incision, the prostatic urethra and bladder were revealed. A 3-0 polypropylene non-absorbable suture was tied around the proximal urethra with a 4F urethral catheter to initiate the obstruction in the anesthetized rats. Following the suture was guaranteed, the catheter was carefully removed, and the incision was closed in layers. Sham rats underwent similar procedures without placing a ligature around the urethra.

### Metabolic Cages

Metabolic cage experiments were conducted 6wk after obstruction to record total water intake and the total amount of urine. Rats were placed in a metabolic cage with free access to water, and food and the voided urine was collected for 24h. Total water consumed and the total amount of urine were noted (14).

### Organ Bath Experiments

Six weeks after the surgery, the rats were killed under anesthesia(ketamine/xylazine; 100/10mg/kg, ip), and bladder tissues were removed for organ bath experiments. Following the removal of connective tissues, the bladder was cut into strips(2×10 mm) along the longitudinal axis isolated from the posterior face to perform *in vitro* functional studies. The strips were mounted under a resting tension(1g) in an organ bath including Krebs solution with 95%O2/5%CO2 at 37°C. The isolated strips were attached to a metal hook and a force transducer. For electrical field stimulation(EFS), an electrical pulse (5ms pulse width, amplitude 90V) was delivered for 15 seconds at increasing frequencies (1- 40Hz) using two platinum electrodes(Grass Instruments, Quincy, MA, USA). After equilibration time (1hr), bladder strips were contracted with 60mmol/L KCl and, cumulative concentrations 10^−7^−10^−4^M of carbachol, EFS(1–40Hz), and ATP(0.1-1mM). The maximum force of KCl depolarization was taken as 100%, and the contractile response was standardized to a percentage of this value(14).

### Western Blot and Quantitative Analysis

Western Blot analysis was performed based on previous studies(14, 15). Approximately half of the bladders were homogenized in RIPA with a protease inhibitor cocktail (Cell Signaling Technology, MA, USA). After centrifugation, total protein quantification was performed by the bicinchoninic acid method (BCA). Equal amounts(40 μg) of protein were fractioned on 10% sodium dodecyl sulfate-polyacrylamide gel and then transferred onto polyvinylidene difluoride membranes for 1h at 100V. The membrane was incubated with blocking solution and then probed with 1:1000 diluted primary antibodies, including endothelial and neuronal NOS (eNOS; 610297 and nNOS; 610308) [BD Transduction Labs, CA, USA], inducible NOS (iNOS; sc-7271), CSE (sc-374249), CBS (sc-133154) [Santa Cruz Biotechnology, Dallas, TX, USA], 3-MST (NBP1-82617)[Novus Biologicals, Littleton, CO, USA], nuclear factor kappa B(NF-κB, 8242), hypoxia-inducible factor 1 alpha (HIF-1α, 36169), and β-actin (4970) [Cell Signaling Technology] at 4°C overnight. Following incubation with secondary antibodies, the visualization of protein bands was performed by a chemiluminescence substrate (Merck, Darmstadt, Germany) and Odyssey Fc system(LI-COR Biosciences, Lincoln, USA). The intensity of protein bands was quantified by Image J software(National Institutes of Health, Bethesda, Maryland, USA).

### Determination of H_2_S and malondialdehyde (MDA) levels in the bladder

H_2_S and MDA levels in bladder tissues were assessed with commercial kits[H_2_S assay kit (kit code: E-BC-K355-M; Elabscience Biotechnology Co.Ltd, Wuhan, China) and MDA assay kit(kit code:700870; Cayman Chemical, Ann Arbor, MI, USA)] based on the instructions. Approximately half of the bladder was homogenized in RIPA. The homogenate was centrifuged at 10,000g and 1,600g for 10min at 4°C to collect the supernatant. The absorbance at 665nm for H_2_S measurement and 540nm for MDA measurement was calculated via a microplate reader(Thermo Scientific, Waltham, MA, USA). Bladder H_2_S and MDA concentrations are expressed as nmol per mg protein by determining protein concentrations in bladder tissue samples using the BCA assay kit.

### Data analysis

The findings were analyzed by Prism v.4(GraphPad Software, San Diego, CA, USA) and expressed as mean± standard deviation (SD). Multiple groups were compared via a one-way analysis of variance(ANOVA) followed by Bonferroni analysis. The minimum level of significance was set at p<0 .05.

## RESULTS

### Characteristics of animals

There was no difference in body weight between groups (Table-1). The bladder weight of rats with PBOO was considerably greater than controls(p<0.001). Monotherapies reduced increased bladder weight, however, there were no statistical differences in the decrement compared to PBOO rats (p<0.05 vs. controls and p<0.01 vs. controls). The combined therapy with NaHS and tadalafil decreased the bladder weight to control levels(p<0.05 vs. PBOO). There were no significant differences in prostate weight, 24-hour water intake, or urine volume between all groups (Table-1).

**Table 1:** Characteristics of animals in the control, partial bladder outlet obstruction and treated groups at 6 weeks.

	Control	PBOO	PBOO+ NaHS	PBOO+ Tadalafil	PBOO+ NaHS Tadalafil
**Body weight (g)**	430.50±44.38	407.86±26.46	422.83±37.66	412.72±58.02	400.72±22.41
**Bladder weight (g)**	0.21±0.05	0.61±0.22***	0.44±0.22*	0.47±0.22**	0.39±0.12#
**Prostate weight (g)**	0.96±0.19	1.03±0.28	1.03±0.29	0.99±0.25	0.88±0.19
**Total urine volume/24 h (mL)**	16.85±4.45	17.86±6.44	14.66±7.58	13.81±4.60	12.72±4.12
**Total water intake/24 h (mL)**	**37.50±7.27**	**45.33±11.87**	**39.16±12.40**	**46.81±12.3**	**47.27±11.03**

**Abbreviations:** NaHS:sodium hydrogen sulfide and PBOO:partial bladder outlet obstruction. Values are the mean ± SD (control=14, PBOO=15, PBOO+NaHS=12, PBOO+Tadalafil=11; PBOO+NaHS+Tadalafil=11).

* P < 0.05;

** P < 0.01;

*** P < 0.001 vs control group;

# P < 0.05 vs PBOO group.

### Contractile responses of the urinary bladder

**Figure 1:**
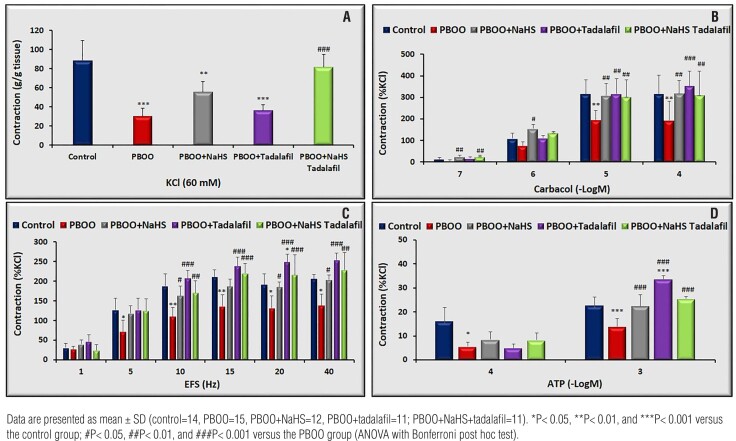
Contractile dose-response curves for A) KCl, B) carbachol, C) electrical field stimulation and D), ATP in bladder strips from all groups.

The membrane depolarizing agent KCl-induced contraction in PBOO bladders was 58% lower than controls(p<0.001),and the combination treatment, but not monotherapies improved this reduction in the obstructed bladder(p<0.001 vs. PBOO, Figure-1A).

Carbachol (10 and 100 μM)-induced contractile responses in obstructed bladder strips were decreased compared to controls(maximal response:51.3%, p<0.01 vs controls). Decreased contractile responses were reversed by combination therapy of NaHS plus tadalafil, NaHS, or tadalafil treatment (Figure-1B).

PBOO decreased EFS-induced contractile response in bladder strips(p<0.05 vs. controls) except at 1Hz frequency. Also, the reduction was normalized by the combination therapy and each monotherapy (Figure-1C).

PBOO induced a considerable reduction in the contractile response to a purinergic agonist, ATP(p<0.001 vs. controls). Contractile responses to ATP(1mM) in obstructed rats receiving tadalafil alone were significantly higher than in control and PBOO rats(p<0.001 vs. controls and p<0.001 vs. PBOO). There were no significant differences in contractile responses to ATP among the combination and NaHS treatment groups (Figure-1D).

### The protein expression of eNOS, nNOS, CSE, CBS, and 3-MST and measurement of H_2_S levels in the bladder tissue

**Figure 2:**
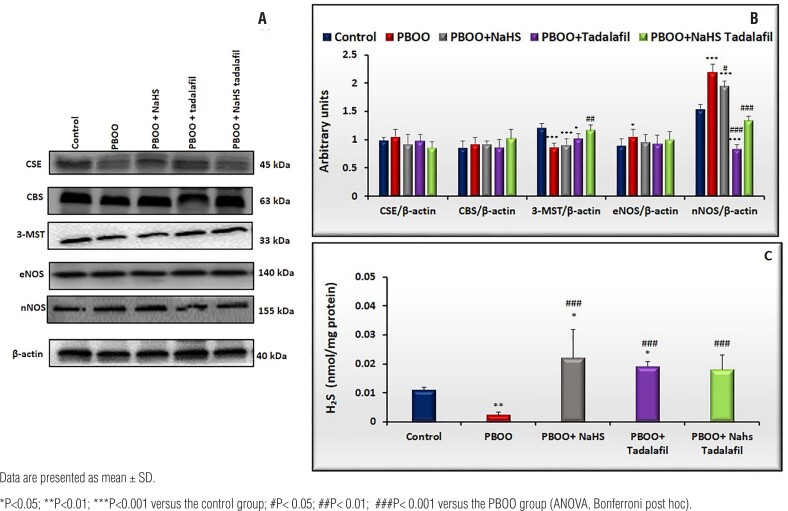
A) semi-quantification of CSE, CBS, 3-MST, eNOS, and nNOS protein expression levels in rat bladder tissues from all groups (control=5, PBOO=6, PBOO+NaHS=6, PBOO+tadalafil=5; PBOO+NaHS+tadalafil=6). B) bar graphs show the ratio of CSE, CBS, 3-MST, eNOS and nNOS protein expression to β-actin expression. C), H_2_S levels in the bladder tissue of rats from all groups (control=5, PBOO=5, PBOO+NaHS=5, PBOO+tadalafil=5; PBOO+NaHS+tadalafil=5).

There was no difference in CSE and CBS protein expression between all groups. 3-MST protein expression was reduced in PBOO rats compared to controls (p<0.001), while was returned after combination treatment (p<0.01 vs. PBOO, Figures 2A and B).

eNOS protein expression in the obstructed bladder was higher than in controls, which was decreased by both monotherapies and the combined treatment (p<0.05 vs. controls, Figures 2A and B).

The protein expression of nNOS was significantly enhanced in the PBOO group and decreased in PBOO with tadalafil treated group(p<0.001 vs. controls), which was reduced by the combined treatment (p<0.001 vs. PBOO Figures 2A and B).

H_2_S levels in obstructed bladders were lower than in controls (p<0.01). The reduction in H_2_S levels in PBOO rats was enhanced by the combination treatment(p<0.001 vs. PBOO) and to a greater extent by NaHS(p<0.05 vs. controls; p<0.001 vs. PBOO), or tadalafil (p<0.05 vs. controls; p<0.001 vs. PBOO) treatment alone compared to controls(Figure-2C).

The protein expression of inflammation and hypoxia-related markers (iNOS, NF-κB, HIF-1α) and MDA levels in the bladder tissue

**Figure 3:**
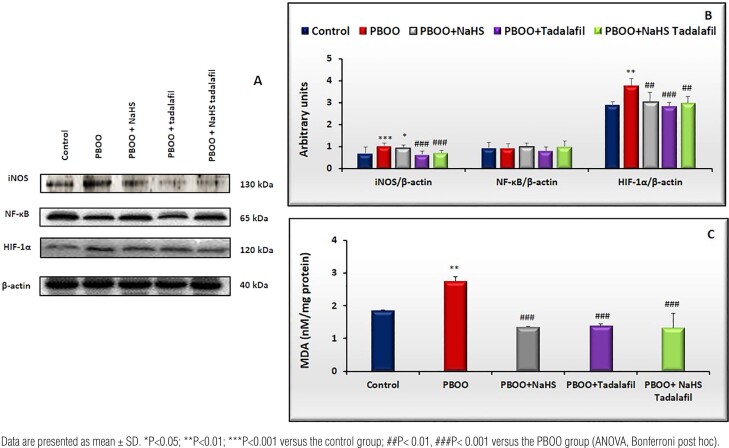
A) semi-quantification of iNOS, NF-_κ_B, and HIF-1_α_ protein expression levels in rat bladder tissues from all groups (control=5, PBOO=6, PBOO+NaHS=6, PBOO+tadalafil=5; PBOO+NaHS+tadalafil=6). B) bar graphs show the ratio of iNOS, NF-_κ_B, and HIF-1_α_ protein expression to _β_-actin expression. C) MDA content in the bladder tissue of rats from all groups(control=5, PBOO=5, PBOO+NaHS=5, PBOO+tadalafil=5; PBOO+NaHS+tadalafil=5).

iNOS protein expression was increased in the obstructed group (p<0.001 vs. controls), while was diminished by tadalafil alone and combination treatment (p<0.001 vs. PBOO, Figures 3A and B).

NF-κB protein levels did not alter in all groups(Figures 3A and B).

HIF-1α protein expression in PBOO bladders was greater than in control bladders(p<0.01), which was reduced by both monotherapies and the combined therapy(p<0.01 vs. PBOO, Figures 3A and B).

MDA levels in PBOO rats were 1.2-fold higher than in controls (p<0.01), which was prevented by all treatments (p<0.001 vs. PBOO, Figure-3C).

## DISCUSSION

The current data confirmed that: (a)carbachol-, EFS- and ATP-caused contraction were lower in the obstructed group than in the control group, and the reduction in responses was improved by the combined treatment, (b)the co-administration of NaHS and tadalafil completely reversed PBOO-induced decrease in 3-MST levels and increase in eNOS, nNOS, iNOS and HIF-1α expression in bladder tissues, and (c)decreased H_2_S levels and raised MDA levels in bladder tissue samples were normalized by the combination treatment.

The obstruction led to an increase in bladder weight compared to the sham-operated group, similar to previous data (16). In the present study, the augmentation in bladder weight was moderately restored in rats receiving monotherapy, when the combination treatment returned this increase. A previous study showed partial improvement in PBOO-induced increase in bladder weight after tadalafil treatment (16). Another study demonstrated that long-term tadalafil treatment prevented functional and histological alterations even though did not reduce bladder weight in the obstructed rat bladder (3). However, there are no previous data to assess the impact of NaHS on the obstruction-caused increase in bladder weight. Previous data indicated that NaHS dose-dependently induced relaxation in the rat bladder (5), and the relaxation was impaired by hypertension and aging-induced decreased H_2_S levels (17, 18). In addition, there is an interaction between NO and H_2_S signaling systems. Sildenafil increased levels of H_2_S in the human bladder (7), and H_2_S enhanced eNOS phosphorylation and xanthine oxidase activity, caused NO production and enhanced NO bioavailability in endothelial cells (19, 20). So, it can be indicated that the combination treatment with NaHS and tadalafil causes additive effects to reduce bladder mass in PBOO rats when compared with monotherapy.

In the present study, urethral ligation reduced in vitro contraction responses in detrusor smooth muscle to carbachol, EFS, ATP and KCl. Furthermore, in the current study, tadalafil treatment partially prevented reduced contractile responses in the obstructed bladder, similar to an earlier study (3). On the other hand, decreased contractility of isolated bladder strips was considerably potentiated by NaHS alone. The previous data showed that PBOO caused a decrease in several parameters of detrusor contractility (14, 21). There was a high correlation between increasing obstructed bladder weight and decreased contractile responses (22). Contrary to our findings, the contractility to carbachol stimulation of PBOO bladder was increased in both rats and human bladder (9). After obstruction, the bladder undergoes a ‘compensated stage’ or ‘decompensated stage’, and the contractile response changes accordingly. In the compensated state, the detrusor muscle undergoes hypertrophy and becomes hypercontractile (23). However, in the decompensated stage, the bladder becomes unable to properly compensate with decreasing bladder contraction (24). The difference between these results could be associated with the obstructed bladders being obtained in different stages of compensation/decompensation (9). A reduction in detrusor contractility in isolated bladders is a characteristic feature of bladder decompensation (25). It can be suggested that PBOO can cause underactive bladder symptoms together with decreased contractile responses of bladder strips.

In our study, CSE and CBS protein expressions did not differ between all groups. Protein expression of 3-MST in the bladder of PBOO and mono-treated groups was lower than in the control group, which was improved by NaHS plus tadalafil treatment. Furthermore, H_2_S concentration in obstructed rat bladder was lower than in controls, which was reversed by combined treatment. Surprisingly, each monotherapy induced significantly higher levels of H_2_S than controls. In a previous study, Fusco et al. (7) demonstrated that sildenafil increased H_2_S levels in the human bladder through the activation of both CSE and CBS enzymes, and the combination of CBS and CSE inhibitors significantly reduced relaxant response to sildenafil in the human bladder (7). It seems that there is a connection between PDE5 activity and the H_2_S pathway in the regulation of bladder function.

NO has a crucial physiological role in the proliferation and differentiation of several cell types and can act as a neurotransmitter in the urothelium and affect bladder function (3, 26). However, the relation between the expression levels of NOS isoforms in bladder function remains unclear. In this study, eNOS and nNOS protein expression were greater in PBOO bladders than in controls. Numerous previous animal studies have demonstrated that the NOS expression increased in the rat obstructed bladder (9, 27), which is responsible for bladder dysfunction. In our study, this increase in nNOS and eNOS expression was further normalized by the combined treatment. It can be suggested that PBOO increased the production of NO by upregulating eNOS and nNOS, but the combination therapy may have a compensatory role for NOS expression in obstruction-induced bladder dysfunction.

In our data, NF-κB protein expression did not differ in all groups. Also, an increment in iNOS protein expression and MDA levels was observed in the obstructed rat bladder. Similarly, previous studies demonstrated no significant differences in total protein expression of NF-κB between the control and obstructed rat bladders (14), and iNOS expression and MDA levels were significantly enhanced by PBOO in rat bladder tissue (28). In addition, iNOS and MDA levels returned to control levels by combination with NaHS and tadalafil in the present study. In an animal model for metabolic syndrome, tadalafil decreased the mRNA levels of inflammatory, pro-fibrotic, and hypoxia indicators in the bladder of rabbits (29). Moreover, treatment with NaHS decreased the renal ischemia/reperfusion-caused overexpression of iNOS and lowered levels of MDA (30).

The protein expression of HIF-1α, a marker of hypoxia, was significantly enhanced in obstructed rats compared to controls, which was normalized by all treatments. PBOO leads to the upregulation of HIF-1α protein expression in the bladder (31). Previous studies showed that the protein expressions of HIF-1α decreased by NaHS in the PBOO rat penis and tadalafil in the PBOO rat bladder (13, 15). The combination of H_2_S donor and PDE5i can recover PBOO-induced bladder dysfunction via decreasing oxidative stress and hypoxia.

The limitations of the study regarding some features of experimental design could affect the interpretation of the outcomes. One of the limitations of the current study is the lack of control treatment groups. Therefore, without these groups, it may be difficult to know if the results were from balanced effects or if the treatments prevented the negative effects of the obstruction. Previous studies demonstrated that NaHS and tadalafil treated control rats displayed similar responses in bladder function, inflammation and oxidative stress markers compared to control rats (32, 33). The current use of a sulfide salt, NaHS is likely to be regarded as a limitation for the application of our study to urological practice, but numerous studies have revealed that H_2_S is effective on bladder function (5, 34). We have shown, for the first time, that the combined treatment with H_2_S and tadalafil is likely to remedy PBOO-induced bladder dysfunction. As obstruction can lead to bladder dysfunction, H_2_S donors and PDE5i may be a new treatment strategy for improving urinary symptoms in BPH patients. The current work supports further clinical studies to focus on developing controllable H_2_S donor drugs, such as AP39, ATB-346 and SG1002, in clinical trials (35, 36).

## CONCLUSIONS

In conclusion, combination therapy involving H_2_S and NO signaling pathways has additive beneficial effects on obstruction-induced bladder dysfunction compared with NaHS or tadalafil alone through an improvement in oxidative stress and hypoxia in the bladder tissue. Collectively, our data suggest that the combination of H_2_S donors and PDE5i might contribute to the improvement of urinary symptoms in BPH patients.
